# Vitamin-D Deficiency and Supplementation Altered the Network of the Coronary Arteries in a Rodent Model—In Situ Video Microscopic Technique

**DOI:** 10.3390/nu14102041

**Published:** 2022-05-13

**Authors:** Hicham Dalloul, Tobias Hainzl, Anna Monori-Kiss, Leila Hadjadj, György L. Nádasy, Marianna Török, Szabolcs Várbíró

**Affiliations:** 1 Károly Rácz Doctoral School of Clinical Medicine, Semmelweis University, Üllői Street 26, H-1085 Budapest, Hungary; 2 Department of Obstetrics and Gynecology, Semmelweis University, Üllői Street 78/a, H-1082 Budapest, Hungary; hainzltobias@gmail.com; 3 Department of Translational Medicine, Faculty of Medicine, Semmelweis University,Üllői Street 78/a, H-1082 Budapest, Hungary; anna.monorikiss@gmail.com (A.M.-K.); leila.hadjadj@gmail.com (L.H.); 4 Department of Physiology, Faculty of Medicine, Semmelweis University, Tűzoltó Street 37-47, H-1094 Budapest, Hungary; nadasy.gyorgy@med.semmelweis-univ.hu; 5 Workgroup for Science Management, Doctoral School, Semmelweis University, Üllői Street 22, H-1085 Budapest, Hungary

**Keywords:** vitamin-D deficiency, network, left-anterior-descending coronary arteries, LAD, cardiovascular disease, video-microscopic technique

## Abstract

The aim of our study was to identify whether vitamin-D deficiency (VDD) can alter the geometry of the coronary-resistance-artery system. Male Wistar rats were divided into vitamin-D-deficient (VD−, n = 10) and vitamin-D-supplemented (VD+, n = 8) groups. After eight weeks, branches and segments of the left-anterior-descending-coronary-artery (LAD) network were analyzed by a video-microscopy technique. Segments were divided into 50 μm-long cylindrical ring units. VDD did not increase the number of morphological abnormalities. The number of segments did not differ between the groups (VD−: 210 and VD+: 224; pooled data of 8 networks). A larger lumen area of branches was found in VD+ group, while 1–4-order branches were lengthier in the VD− group. VD− rats had less rich coronary-resistance-artery networks in terms of 50 µm-long units. (VD−: 6365 vs. VD+: 6602; pooled data of 8 networks). VD+ animals were richer in the 100–350 µm outer diameter range, and VD− animals were richer in the 400–550 µm-diameter units. In VD− rats, 150–200 and 300 µm units were almost missing at higher flow distances from the orifice. Serum vitamin-D alterations caused by dietary changes can affect the geometry of the coronary-artery network, which may contribute to vitamin-D-dependent changes in cardiovascular mortality.

## 1. Introduction

It has been a hundred years since McCollum first used the term ‘vitamin D’ in 1922 [1]. Since then, vitamin D has been known as a regulator and key molecule of calcium metabolism, serum calcium levels, and bone mineralization in the human body [2,3,4,5]. Beyond its skeletal effects, the role of vitamin D has been confirmed in numerous different biochemical processes and diseases including but not limited to mental and psychological disorders [6], cancer [7], immunological aspects [8], as well as pregnancy and neonatal outcomes [9]. Therefore, some consider it as a general physiological regulatory molecule.

Its role in the cardiovascular system is being extensively studied. Observational studies have confirmed the linkage of vitamin-D deficiency with hypertension and cardiovascular-related deaths [10,11]. As an explanation for this, the vitamin-D receptor (VDR) has been found to be widely distributed in the cardiovascular-system cells. However, the role of vitamin-D supplementation in reducing cardiovascular harm is unclear based on the results of comprehensive studies. Most comparative studies leave the question open [12,13,14,15,16,17,18,19]. However, there are studies that confirm [20] and some that do not support [21,22] the role of vitamin-D supplementation in reducing the cardiovascular mortality rate.

One of the most studied cardiovascular consequences of vitamin-D deficiency is its role in myocardial ischemia [23]. There are several mechanisms that can contribute to this, all of which can be affected by vitamin-D deficiency. Most studies have focused on the effect of vitamin D on different cell types (e.g., cardiomyocytes or vascular smooth-muscle cells) [14,24]. Arterial stiffness [25], altered endothelial function [26,27], increased atherogenesis [28], and changes in oxidative-stress tolerance [29] can all play a role in it. The topic of altered vascular contractility and relaxational ability associated with vitamin-D deficiency was studied by our research team in a rodent model in coronary arteries [30,31], cerebral arteries [32,33], renal arteries [34], carotid arteries [35] and the aorta [36].

The examination of the geometry of the vascular network, the description of the hemodynamically advantageous course, and branches of the vessels can be traced back to the rules described by Murray [37,38]. Although it has been known for almost one hundred years that deviations from this law reduce the efficiency of circulation, most of the research on vascular examination has focused on the histology, biochemical properties, and contractility of blood vessels. The development of the micropreparation technique of intramural coronary-resistance-artery networks in the rodent model [39,40] and the increasingly widely used technique of video microscopy have made it possible to study the vascular-network system in its complexity and to observe the effect of different variables on the whole network. Our research team successfully demonstrated the effect of hypertension, aging, and training on the entire geometry of the coronary network [39,41,42] using this technique.

In our study we sought the answer to how vitamin-D deficiency and the biochemical processes influenced by it can change the geometry, branching, and distribution of arteries of different diameters of the left-anterior-descending-artery (LAD) network. Our hypotheses were: (1) vitamin D does not only affect the histological structure and contractile function of the coronaries, but also plays a role in forming the geometry of the entire network, thus gaining a potential role in cardiovascular mortality; (2) the lack of vitamin D, which is known to be involved in angiogenesis, may produce hemodynamically disadvantageous network anomalies in the coronary network; (3) that changes may occur in the location of the vascular-network unit population due to vitamin-D deficiency and supplementation.

## 2. Materials and Methods

### 2.1. Ethical Approval and Animals

The study was designed and performed based on the Guide for the Care and Use of Laboratory Animals published by the US National Institutes of Health (8th edition, 2011) and the European Union (Directive No. 2010/63/EU). All procedures were approved by the Ethical Committee of Hungary for Animal Experimentation and University authorities (permission number: IRB: 8/2014 PEI/001/1548-3/2014, PEI/001/820-2/2015). Four-week-old male (n = 18) Wistar rats (Semmelweis University in agreement with Charles River LTd., AnimaLab, Vác, Hungary) were randomly divided into two experimental groups: a group with vitamin-D deficiency (VD−, n = 10) and a group with vitamin-D supplementation (VD+, n = 8).

### 2.2. Chemicals

Vigantol oil (20,000 IU/mL cholecalciferol suspension) was provided by Merck/Merck Serono (Darmstadt, Germany). For ex vivo video-microscopic analysis, specimens were immersed in normal Krebs–Ringer solution (nKR), which consisted of the substances published in [41]. The substances used for the nKR were obtained from Reanal (Budapest, Hungary) and Sigma-Aldrich (Sigma-Aldrich, St. Louis, MO, USA–Budapest, Hungary).

### 2.3. Chronic Treatment of the Rats

During the 8-week-long chronic-treatment period, rats were housed in constant environmental conditions (relative humidity (40–70%), constant room temperature (22 °C ± 1 °C) and light–dark cycle (12 h each)). The animals were provided with different laboratory rat chow (with different composition, see later) and tap water ad libitum according to the following group protocols. Vitamin deficiency was induced by a vitamin-D-free diet (<5 IU/kg Vitamin D3, Vitamin D Free Lab Rat/Mouse Chow, Ssniff Spezialdiäten GmbH, Soest, Germany) for 8 weeks (the average 25-OH-D3 level at the end of chronic treatment: 3.59 ± 0.21 ng/mL) [32,43]. Animals of the VD+ group were fed a standard laboratory diet (1000 IU/kg of Vitamin D) for 8 weeks. Oral administration (through a gavage cannula) of additional vitamin D was given as follows: 500IU cholecalciferol on week 2, and weeks 4–8, a weekly dose of 140 IU/100 g (the average 25-OH-D3 level at the end of chronic treatment: 19.66 ± 0.81 ng/mL) [32,43]. No unexpected medical condition, complication or side effect was observed during the treatment period.

### 2.4. Preparation and Recording of LAD Coronary Networks

After chronic treatment, the preparation of coronary-resistance-artery networks from the heart and in situ video-microscopy recording during perfusion were performed as previously described [40]. In brief, after anesthesia (Nembutal, 45 mg per kg, intraperitoneal), the heart was removed and the LAD coronary-artery network was prepared by careful microdissection in cold Krebs–Ringer solution under high magnification [40]. With this technique, the segments of the LAD remained intact and branches larger than 80 µm became visible. After cannulation of the orifice, the network of the LAD was perfused with nKR solution (pH = 7.4, 37 °C, bubbled with O_2_ 20%, CO_2_ 5% and N_2_ 75%) at close to in vivo pressures. After a few minutes of equilibration, the coronary network was measured by a video microscope using different magnifications (low and high magnifications, 8.58 and 1.47 µm/pixel). For accurate geometric reconstructions of the networks, low- and high-magnification images were photographed and then analyzed off-line (ImageJ software, NIH, Bethesda, MA, USA) as previously described [39]. The pixel µm calibration was performed using a micrometer etalon (Wild, Heerbrugg, Switzerland).

### 2.5. The Coordinate System and Geometric Analysis

**Figure 1:**
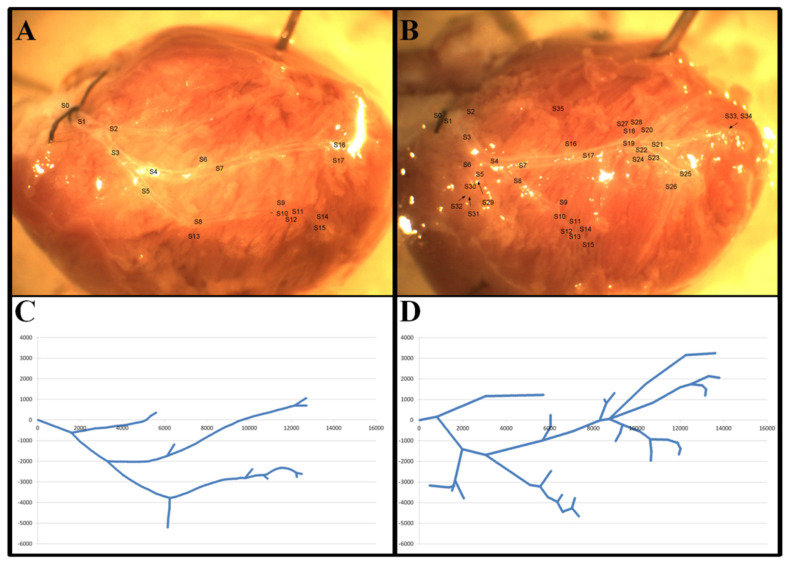
Representative video-microscopic images of segmental and branching analysis (**A**,**B**) and mapping of the LAD networks in a coordinate system, following 50 μm-ring-unit analysis (**C**,**D**). Note the difference between the two experimental groups in the network density of the LAD coronary artery, shown in the image and the associated coordinate system of a typical vitamin-D-deficient (**A**,**C**) and a typical vitamin-D-supplemented animal (**B**,**D**).

Good-quality low- and high-magnification pictures taken from perpendicular position were selected to rebuild a horizontally stretched network for analysis. All bifurcations and segments of the coronary network were then marked in the >80 μm range. A coordinate system was created based on the high-magnification pictures of the networks as previously described [39]. In brief, the X-axis was created between the orifice and the apex of the heart. The Y-axis was erected perpendicular to the X-axis, with positive values in the direction of the left ventricle. The zero point for both axes was the orifice. Segment lengths and bifurcation angles were measured, images of segments were divided into 50-micrometer-long cylindrical units, and the diameter, direction and coordinate position of all components were determined as shown in Figure 1.

### 2.6. Segment Analysis

The whole network was divided into segments at bifurcations and the segments were then numbered. Although the diameter of the vessel generally does not change along the segment, outer and inner vessel diameters were also measured at three points along the segmental axis. Distances of the branching points from the origin, angles of the segmental axes of two related segments, and angles with the coordinate were also analyzed. For each network component (bifurcations, segments, ring units), the direct distance from the orifice was computed using the coordinates. In addition, for each segment, a length for the potentially curved axis was also computed. This way “flow distances” from the orifice or for the segments could be compared with direct distances, giving an opportunity to calculate the tortuosity of the network. Segmental analysis was performed by counting the number of segments and measuring their length, as well as the outer diameter and wall thickness. From the inner-radius (r_i_) data, the lumen cross-section area was calculated according to the following formula: Lumen cross-section area (µm^2^) = r^i2^ × Π.

### 2.7. Branching Analysis

All branches were identified and analyzed as previously described [39]. All bifurcations were sorted into dichotomic, multiplex or lateral branching categories. All bifurcations were tested for the validity of Murray’s law: D_om_^3^ = D_od1_^3^ + D_od2_^3^, where D_o_ is the outer diameter in µm, and _m_, _d1_ and _d2_ are the mother and daughter branches, respectively. The asymmetry index (A_i_) was calculated according to the following formula: A_i_ = D_od1_/D_od2_, where Do is the outer diameter in µm, and _d1_ and _d2_ are the daughter branches (the data of the larger daughter branch were always placed in the numerator/top).

### 2.8. Analysis of 50 µm-Long Vascular Ring Units

Theoretically, all coronary-artery networks were divided into 50 μm-long ring units as a base unit of the network as previously described [39]. The ring units were located in the X–Y coordinate system. The outer and inner diameters, wall thickness, X and Y coordinates for the ring-unit center, angle of axis with the X-axis, flow distance, and the direct distance from the orifice were measured. The ring-unit analysis was performed by constructing lists of ring units in a certain inner/outer-diameter range and at a certain direct/flow distance from the orifice.

### 2.9. Network Anomalies

In addition to the measurable parameters of the networks, other hemodynamically significant alterations were also recorded. Parallel-running branches, broken courses, and multiple branching (e.g., triple) present in the systems were counted. Tortuosity was measured. Tortuosity (T), curvature, and ratios of segments were computed by comparing the direct distance between the start and end points of the segment as well as the potentially curved length of the segment’s axis following the route of blood flow: T(%) = 100 − (segment length in the airline (µm) × 100/segment length in real (µm)).

### 2.10. Statistical Analysis

GraphPad Prism 5, SPSS Sigma Stat and Excel software were used for statistical analysis. All data are presented as mean ± SEM. In the case of normal distribution (tested using the Shapiro–Wilk method), the two-tailed unpaired Student’s *t*-test was performed. The Mann–Whitney test was performed in the case of non-normal distribution (in case of ‘Flow lengths of the segment’ and ‘Wall thickness of 50 µm ring units as a function of different diameter ranges’). Morphological abnormalities were counted, pooled and normalized in 8 rats for all perfused networks. Their number was determined by the test. Frequencies of ring units in different diameter ranges in VD+ and VD− coronary networks were compared with the χ^2^ test. The Pearson correlation method was used to evaluate the interconnection between bifurcation asymmetry and angle. A 3D scatter plot was used to show differences in bifurcation branch angle as a function of vessel diameter. The level of deviation of the flow route from the direct distance from orifice and the diameter of the ring were analyzed on 3D plots of two-dimensional histograms. The number of ring units in a given diameter and flow-distance range was analyzed in two-dimensional histograms and visualized in 3D (color-coded) maps. *p* < 0.05 was used as the criterion for statistical significance.

## 3. Results

### 3.1. Body-Weight, Heart-Weight and Blood-Pressure Data

There was no significant difference between the VD− and VD+ groups in terms of body weight (VD−: 481 ± 15 vs. VD+: 477 ± 19 g) or heart weight (VD−: 1.37 ± 0.04 vs. VD+: 1.32 ± 0.08 g). The measurements of the mean blood pressure through cannulation of the right carotid artery showed no difference between the two groups (VD−: 95.39 ± 4.35 vs. VD+: 88.18 ± 6.57 mmHg, animals in Nembutal anesthesia).

### 3.2. Segment Analysis

**Figure 2:**
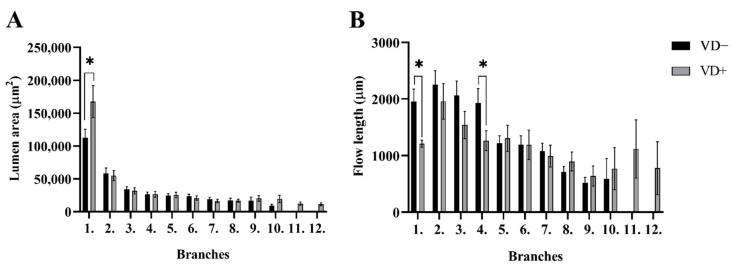
Segments analysis. (**A**) Lumen area of the segments from the VD− and VD+ animals. The lumen area of the first-order branches was significantly larger in VD+ group. (**B**) Flow lengths of the segments from the VD− and VD+ animals. The 1st- and 4th-order branches were lengthier in VD− rats. Values are means ± SEM. Two-tailed unpaired Student’s *t*-test and Mann–Whitney-test. * *p* < 0.05 VD− vs. VD+.

The number of coronary-resistance-artery segments in the subsurface network, down to an outer diameter of 80 micrometers, did not differ between the two groups (VD−: 210 and VD+: 224 segments; pooled, normalized data of 8 networks). One characteristic difference was the significantly larger lumen area of the main (first-order) branches in VD+ group. The lumen diameters did not differ during the following branching steps (Figure 2A). Additionally, 1–4-order branches were lengthier in VD− rats (significant with the paired *t*-test for 1st- and 4th-order branches, (Figure 2B). It is important to note that 11–12-order branches were found only in the VD+ group in the subsurface network (Figure 2).

### 3.3. Branching Analysis

**Figure 3:**
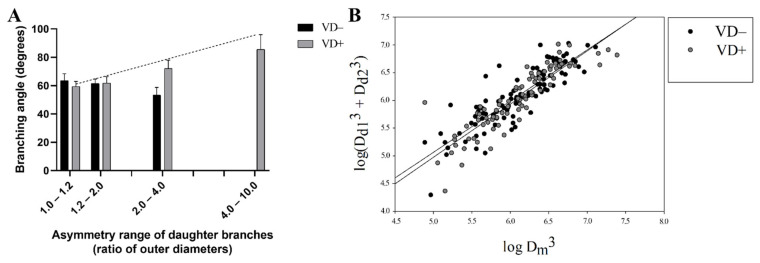
Analysis of branching. (**A**) Asymmetry range of daughter branches (ratio of outer diameters). The asymmetry index (with increasing branching angle) was significantly elevated only in VD+ groups (with Pearson correlation, *p* < 0.05). (**B**) The Murray law. Branches are obeying the Murray law.

One characteristic of microvascular bifurcation geometry is that larger daughter branches tend to deviate less from the axis of the mother branch than smaller daughter branches. As a result, the branch angle between daughter branches increases with the increasing asymmetry index. In substituted networks it is duly seen (Figure 3A; significant with the Pearson correlation). Such a linkage is clearly missing in the vitamin-D deficient animals (Figure 3A).

Another characteristic of microvascular bifurcation geometry is that the lumen diameters of daughter branches obey the Murray law. Figure 3B demonstrates that bifurcations of both groups fairly adhered the Murray law (scatter from the expected X = Y line was almost equal, n.s. with the F probe).

### 3.4. Abnormalities

Vitamin-D deficiency did not elevate the number of such morphological network abnormalities as parallel running, broken courses, multiple branching, or tortuosity (Table 1).

### 3.5. Vascular Ring-Unit Analysis

**Figure 4:**
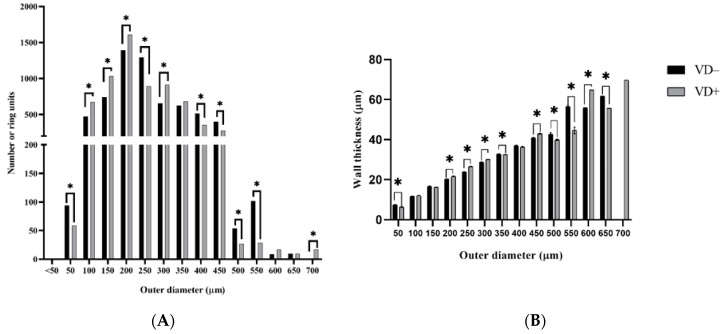
Analysis of ring units. (**A**) Number of 50 µm ring units as a function of different diameter ranges. In VD− networks, the number of rings decreased in the range of 100–300 µm (except at 250 µm), and the number of rings increased in the range of 400–550 µm. Normalized in 8/8 rats. Significantly different with the Chi-probe (*p* < 0.05). (**B**) Wall thickness of 50 µm ring units as a function of different diameter ranges. Wall thickness was increased in the 50, 350, 500, 550 and 650 µm range in VD− group. However, the wall thickness was bigger in the 200, 250, 300, 450 and 600 µm range in VD+ group. Values are means ± SEM. Mann–Whitney-test. * *p* < 0.05 VD− vs. VD+.

VD− animals had somewhat less rich coronary-resistance-artery networks than VD+ rats. (Figure 1) When the whole network was divided into 50 µm-long units, they had a significantly lower number of such units (6365 vs. 6602; pooled, normalized data of 8 animals; *p* < 0.0374 with the χ^2^ probe). Figure 4A demonstrates that this elevated number of vascular units in VD+ animals was present throughout the 100–300 µm outer diameter range. The only exception is at 250 µm, the maximum of the histogram in VD− animals pushed upward from the 200 µm of the substituted animals.

However, VD− animals were richer in larger-diameter units (400–550 µm). In practically the same large-diameter group, a thickening of the wall was also demonstrated with an opposing alteration in the most frequent 200–300 µm diameter range (Figure 4B).

**Figure 5:**
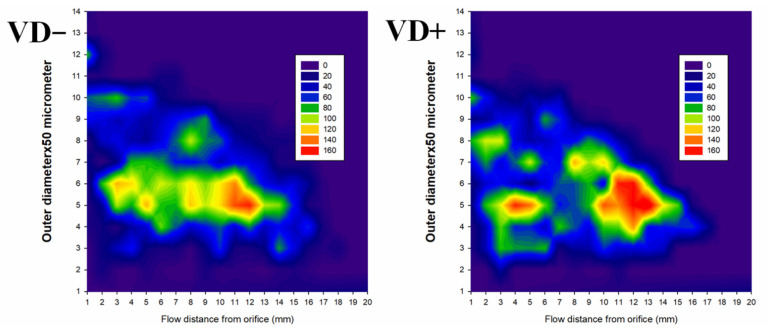
Frequency of ring units (color-coded) for different diameters and flow distances from the orifice. Note that in VD− rats a new population of 250 µm units appears at a 6–9 mm flow distance from the orifice, while at the same locations there is a diminishment of 350 µm units. In VD− rats, 150–200 and 300 µm units are almost missing at 10–15 mm flow distances.

The next question is at what location of the network such vascular unit-population changes do occur. The two-dimensional histograms of Figure 5 demonstrate that in vitamin-D-deficient rats, a new population of 250 µm units appears at a 6–9 mm flow distance from the orifice, while at the same locations there is a diminishment of 350 µm units. In vitamin-D-deficient rats, 150–200 and 300 µm units are almost missing at 10–15 mm flow distances.

## 4. Discussion

Our first studies on the whole network geometry of in situ perfused coronary-artery networks in Vitamin-D-deficient and substituted animals demonstrated that chronic vitamin-D deficiency induces characteristic changes in network geometry. In the present study, we first demonstrated that vitamin-D deficiency affects cardiac-tissue perfusion without abnormalities in coronary branching patterns that modulate cardiac hemodynamics. The major finding of our investigation can be summarized as follows: (1) VDD did not increase the number of morphological abnormalities; (2) VDD resulted in a less rich coronary-resistance-artery network; (3) VDD resulted in 150–200 and 300 µm units that were missing at higher flow distances from the orifice; (4) in contrast, vitamin-D supplementation resulted in a richer network; (5) vitamin-D supplementation resulted in a larger lumen area of the branches, the branching pattern was optimal, and abnormalities did not increase.

It is a known fact that vitamin-D deficiency increases the risk of cardiovascular events, although vitamin-D supplementation does not clearly balance this effect, as demonstrated by several meta-analyses [10,11,12,14,17,18,20,21,22]. Vitamin-D-deficiency-induced cardiovascular risk is associated with a combination of several factors [44]. Low vitamin D has been shown to be associated with high blood pressure [45,46] obesity [47,48,49,50], insulin resistance [51,52], and dyslipidemia [53]. In several studies, a description of normal 25-OH-D3 levels can be found. Trechsel et al. published values of 40 nmol/L (16 ng/mL) in animals consuming a normal vitamin-D diet [54]. At the recommended daily intake (values projected onto human model: 0.015 mg/day, which corresponds 300 E/day), Mirhosseini et al. achieved vitamin-D levels of 17.2 ng/mL in Wistar rats over four weeks of treatment.

According to the same article, at a high daily dose of vitamin D (values projected onto human model: 0.25 mg/day, which corresponds 5000 E/day), vitamin-D levels of 43.2 ng/mL were achieved in Wistar rats over four weeks of treatment, while a vitamin-D-free diet resulted in 12 ng/mL [55]. Wilson et al. reported levels of 37–38 ng/mL of 25-OH-D3 but also used vitamin-D supplementation in addition to the standard diet [56]. These values were not approached in any of the groups.

For extremely low ranges, Treschel considered a vitamin-D deficiency below 25 nmol/L (10 ng/mL) [54], which we have clearly achieved in our animal model. The definitively toxic concentration is 360 ng/mL based on Takács et al. [57] and 224 ng/mL according to Mirhosseini et al. [55]. Such toxic values were not achieved in any of the groups of our study.

According to Halloran’s work, vitamin-D levels of 14-day-old breastfed Holtzman rats whose mother received 25U daily vitamin-D supplementation was 10 ng/mL. A vitamin-D level of 8 ng/mL was found after weaning at the age of 25 days, and 9 ng/mL was found in animals three weeks after being weaned from breast milk and fed with normal laboratory formula during this period [58]. Vitamin-D levels in Holtzman rats that had just been weaned in Weishaar’s work were below the measurement range and remained there after a nine-week vitamin-D-free diet, while it increased to 9.5 ng/ml after six weeks and 14.1 ng/mL after nine weeks with vitamin-D supplementation of 30 E/day [59].

Thus, we can suspect that the rat weaned from milk suffers from a relatively moderate vitamin-D deficiency, which we were able to normalize and even slightly exceed the normal level in our model by supplementation. On the other hand, by our model an eight-week vitamin-D-free diet starting from weaning kept vitamin-D levels persistently low. The effects of vitamin-D deficiency on the histological characteristics of vascular cross-sections and the contraction and relaxation properties of coronaries [30,31] has already been demonstrated by our research group.

We found no difference between the body weight of the study groups. Vitamin D also affects the differentiation of myocardial cells and affects myocyte proliferation through its action on myoproliferative genes and the renin–angiotensin system [60,61,62]. However, we found no difference in heart weight between the two groups, making it easier to compare the coronary networks.

For the technical implementation of our study, we chose the micropreparation and video-microscopic analysis of the LAD branch system [39,40,41,42]. The technique allows the whole network to be analyzed in its complexity, using physiological pressure conditions to which the blood vessels respond with their own myogenic tone. This contributes to a proper evaluation of the dimensions of the specific parts of the network involved. The spatial formation of the coronary network ensuring that all parts of the heart muscle tissue receive equal and adequate amounts of oxygen and nutrients. This process is regulated by many factors [63]. The network must be of adequate resistance and hemodynamically advantageous to distribute blood flow to the highly demanding ventricular tissue. The length, the number of segments, geometry and quantity of branches, bifurcations, their potential tortuosity, diameter of the lumen, thickness of the wall, and the distribution of all these parameters will, be among others, a function of the distance from the origin of the network (in that case the coronary orifice). Knowing the effect of vitamin D in the regulation of vascular remodeling [56,57], we could expect that there is a difference between the two groups in the appearance and geometric characteristics of the network as a whole system. A geometric analysis of the network allows us to recognize different anomalies and to analyze the systemic effects of vitamin-D deficiency on vascular-network formation, vascular cross-sectional structure, and changes in the coronary network, possibly contributing to increased cardiovascular mortality.

Basic characteristics of the morphometry of a coronary network and the effect of hypertension have been studied in different models [64,65,66,67]. Based on the work of Murray and Zamir, we can gain insight into the properties of hemodynamically beneficial vascular networks [37,38,68]. Accordingly, we considered multiple branches, branches running in parallel, broken courses, and a high range of tortuosity as disadvantageous patterns requiring higher-than-optimal mechanical energy. Based on previous work by our research group, we have demonstrated that high blood pressure increases the incidence of these anomalies in the rat LAD network. [42] In earlier works, we have found that the Murray law of bifurcations was maintained in cases of hypertension, aging and physical exercise [39,41]. In our present study, there was no significant difference between the two groups in terms of the unfavorable coronary anomalies characterizing aged and hypertensive networks [42]. At the same time in our study, we found no difference between the mean blood pressure of vitamin-D-deficient and supplemented animals. All this suggests that although vitamin D is known to play a role in vasculogenic processes [69,70], it is not vitamin-D deficiency alone that causes vascular-network anomalies, but in the long term it may be caused by high blood pressure due to vitamin-D deficiency. In Weishaar’s previous study [59], calcium, creatin phosphokinase (CPK) and phosphate levels along with blood pressure were examined in young rats as a function of vitamin-D supplementation. It was shown that vitamin-D deficiency significantly increased the blood pressure of the animals between weeks two and six of the diet; however, there was no difference in blood pressure from week seven to nine of the diet. Consistent results were obtained in our present study after eight weeks of treatment. In Weishaar’s study [59], perturbed Ca homeostasis was suggested to be responsible for the very early rise in blood pressure; however, the eight-week diet had no effect on it. On the other hand, recent reports stress the relationship between long-term vitamin-D deficiency and hypertension [10,11,14,45,46]. The atherogenic effect of the vitamin-D deficiency may be responsible for the development of hypertension in the long term [69,70]. According to the rheological principles of Poiseuille, in a hemodynamic system, the length of the vessels is directly proportional, while the fourth power of the lumen diameter and the number of branches connected in parallel are inversely proportional to the resistance of the network. We can suppose that in the vitamin-D-deficient experimental group, the vascular resistance of the coronary arteries might be higher. In our experimental model, coronary-network abnormalities that increased network resistance due to vitamin-D deficiency were expected to result in a long-term increase in blood pressure, ensuring an adequate supply of nutrients to the tissues.

Further analyzing our coronary-artery networks with a focus on branching, we found that more asymmetric bifurcations do not have larger bifurcation angles in vitamin-D-deficient rats as previously expected and as shown in the VD+ group. This can be considered a disadvantageous alteration from the hemodynamic point of view. Vitamin-D deficiency, however, did not affect the regulation of the lumen diameter by shear forces, and the Murray law was strictly maintained in both groups.

Analyzing the coronary-resistance-artery segments in the subsurface network, the number of segments with outer diameters down to 80 micrometers did not differ between the two groups. At the same time, some characteristic difference was found. A significantly larger lumen area of the main (first-order) branches in the VD+ group may allow for greater blood flow. Lumen diameters did not differ during the following branching steps. Additionally, 1–4-order branches were lengthier, and segments formed by them were longer and had thicker walls with reduced cross sections in VD− rats, which may result in greater resistance of those specific segments. It is important to note that 11–12-order branches were found only in the VD+ group. This result objectively confirmed the preliminary observation made during the review of the coronary networks that the LAD networks of the vitamin-D-deficient group were much denser. In further analysis of the network, the coronary network was divided into 50 μm elements and the number of ring elements, diameter, wall thickness, lumen cross section, and the distribution of these ring elements of different thicknesses as a function of distance from the orifice were examined. Although heart masses did not differ, the number of normalized ring elements of the network was significantly increased in the VD+ group, resulting in a richer LAD system.

The less rich network of the vitamin-D-deficient animals was formed by the smaller number of arterioles with diameters of 150–200 µm. On the contrary, the elevated number of larger-diameter (400–550 µm) “small artery” units could not offset this phenomenon.

The extra distance of the individual ring units appeared elevated in the VD+ group. The elevated extra distance has been considered as a hypertensive characteristic so far, mainly based on studies of retinal hypertensive abnormalities [71,72], but no other hypertensive characteristic (hypertension, network anomalies) was found in the VD+ group, so this may contribute to the elevated ring-unit number and the richer network.

The distribution pattern of the ring elements also shows that tissue perfusion may have improved because the number of small- to medium-diameter vessels distant from the orifice was significantly increased in the VD+ group.

The wall thickness was higher in the VD+ group for medium (100–300) diameters, while the elevation of the wall thickness in the VD− group for the smallest (<50 μm) and largest diameters (400–550) could be seen.

The maximum of the diameter frequency histogram of the VD+ group was shifted upward to 250 from 200 µm, which can also be interpreted as a hemodynamically advantageous alteration.

In our earlier studies [29,31] 200-micrometer-diameter rat coronary-resistance arterioles were examined with pressure arteriography. In vitamin-D-deficient rats, a reduced spontaneous tone was found, and in the fully relaxed state the segments had a narrowed lumen and increased wall thickness. In the present work, segmental geometry was studied along the whole network in pressure-perfused preparations where spontaneous tone could develop. The larger wall thickness we measured in the 200–300 µm range in the substituted animals could be the result of this larger myogenic tone.

The limitation of our study is in the case numbers and the animal study; however, it is also a strength that the genetically homogenous background of the animals resulted in highly homologous networks and a definitive causality of the results. Because of these reasons, only the altered vitamin-D levels could have led to the differences in the network structure. We must mention that both the difficulties of the micropreparation technique and the complexity of the statistical analysis of the vessel rings clearly limited the number of individuals that could be tested. More observers could study a larger number of animals; however, it might lead to greater interobserver variability in the evaluation of network anomalies, which should also be avoided.

## 5. Conclusions

The wall-thickness elevation in larger branches, the diminishment of the number of smaller units, and the alterations in the bifurcation angles definitively indicate the involvement of vitamin-D receptors in the morphological formation of coronary-resistance-artery networks. Thus, vitamin-D supplementation improves tissue perfusion due to the richer network. In the richer network, the branching pattern was optimal and abnormalities did not increase. While these can be expected to have only moderate hemodynamic effects, pathological processes can be altered by them.

## Figures and Tables

**Table 1:** Pooled number (normalized in 8/8 rats) of morphological deformities found in the resistance-artery network of the left-anterior-descendent coronary artery of vitamin-D-deficient (VD−) and vitamin-D-supplemented (VD+) groups.

Morphologic Deformity	VD−	VD+	Chi2 (χ^2^) Probe Significance Level
Parallel running	2	3	0.65
Broken course	7	6	0.74
Multiple branching	11	8	0.49
Tortuosity > 8	7	5	0.56
Sum of all deformities	27	22	0.48

## Data Availability

Data are contained within the article.

## References

[1] Mccollum E.V., Pitz W., Simmonds N., Becker J.E., Shipley P.G., Bunting R.W. (1922). Studies on experimental rickets: XXI. An experimental demonstration of the existence of a vitamin which promotes calcium deposition. J. Biol. Chem..

[2] Pike J.W., Christakos S. (2017). Biology and Mechanisms of Action of the Vitamin D Hormone. Endocrinol. Metab. Clin. N. Am..

[3] Montenegro K.R., Cruzat V., Carlessi R., Newsholme P. (2019). Mechanisms of vitamin D action in skeletal muscle. Nutr. Res. Rev..

[4] Gil Á., Plaza-Diaz J., Mesa M.D. (2018). Vitamin D: Classic and Novel Actions. Ann. Nutr. Metab..

[5] Dusso A.S., Brown A.J., Slatopolsky E. (2005). Vitamin D. Am. J. Physiol. Renal. Physiol..

[6] Lerner P.P., Sharony L., Miodownik C. (2018). Association between mental disorders, cognitive disturbances and vitamin D serum level: Current state. Clin. Nutr. ESPEN.

[7] Jeon S.-M., Shin E.-A. (2018). Exploring vitamin D metabolism and function in cancer. Exp. Mol. Med..

[8] Charoenngam N., Holick M.F. (2020). Immunologic Effects of Vitamin D on Human Health and Disease. Nutrients.

[9] von Websky K., Hasan A.A., Reichetzeder C., Tsuprykov O., Hocher B. (2018). Impact of vitamin D on pregnancy-related disorders and on offspring outcome. J. Steroid Biochem. Mol. Biol..

[10] Pilz S., Tomaschitz A., Ritz E., Pieber T.R. (2009). Vitamin D status and arterial hypertension: A systematic review. Nat. Rev. Cardiol..

[11] Kilkkinen A., Knekt P., Aro A., Rissanen H., Marniemi J., Heliövaara M., Impivaara O., Reunanen A. (2009). Vitamin D Status and the Risk of Cardiovascular Disease Death. Am. J. Epidemiol..

[12] Norman P.E., Powell J.T. (2014). Vitamin D and cardiovascular disease. Circ. Res..

[13] Al Mheid I., Patel R.S., Tangpricha V., Quyyumi A.A. (2013). Vitamin D and cardiovascular disease: Is the evidence solid?. Eur. Heart J..

[14] Latic N., Erben R.G. (2020). Vitamin D and Cardiovascular Disease, with Emphasis on Hypertension, Atherosclerosis, and Heart Failure. Int. J. Mol. Sci..

[15] Wimalawansa S.J. (2018). Vitamin D and cardiovascular diseases: Causality. J. Steroid Biochem. Mol. Biol..

[16] Pilz S., Verheyen N., Grübler M.R., Tomaschitz A., März W. (2016). Vitamin D and cardiovascular disease prevention. Nat. Rev. Cardiol..

[17] Kunadian V., Ford G.A., Bawamia B., Qiu W., Manson J.E. (2013). Vitamin D deficiency and coronary artery disease: A review of the evidence. Am. Heart J..

[18] de la Guía-Galipienso F., Martínez-Ferran M., Vallecillo N., Lavie C.J., Sanchis-Gomar F., Pareja-Galeano H. (2021). Vitamin D and cardiovascular health. Clin. Nutr..

[19] Rai V., Agrawal D.K. (2017). Role of Vitamin D in Cardiovascular Diseases. Endocrinol. Metab. Clin. N. Am..

[20] Bouillon R., Manousaki D., Rosen C., Trajanoska K., Rivadeneira F., Richards J.B. (2021). The health effects of vitamin D supplementation: Evidence from human studies. Nat. Rev. Endocrinol..

[21] Manousaki D., Mokry L.E., Ross S., Goltzman D., Richards J.B. (2016). Mendelian Randomization Studies Do not Support a Role for Vitamin D in Coronary Artery Disease. Circ. Cardiovasc. Genet..

[22] Afzal S., Brøndum-Jacobsen P., Bojesen S.E., Nordestgaard B.G. (2014). Genetically low vitamin D concentrations and increased mortality: Mendelian randomisation analysis in three large cohorts. Br. Med. J..

[23] Milazzo V., de Metrio M., Cosentino N., Marenzi G., Tremoli E. (2017). Vitamin D and acute myocardial infarction. World J. Cardiol..

[24] Lin L., Zhang L., Li C., Gai Z., Li Y. (2019). Vitamin D and Vitamin D Receptor: New Insights in the Treatment of Hypertension. Curr. Protein Pept. Sci..

[25] Chen N.-C., Hsu C.-Y., Mao P.C.-M., Dreyer G., Wu F.-Z., Chen C.-L. (2019). The effects of correction of vitamin D deficiency on arterial stiffness: A systematic review and updated meta-analysis of randomized controlled trials. J. Steroid Biochem. Mol. Biol..

[26] Kim D.-H., Meza C.A., Clarke H., Kim J.-S., Hickner R.C. (2020). Vitamin D and Endothelial Function. Nutrients.

[27] Napoli C., de Nigris F., Williams-Ignarro S., Pignalosa O., Sica V., Ignarro L.J. (2006). Nitric oxide and atherosclerosis: An update. Nitric Oxide.

[28] Bennett A.L., Lavie C.J. (2017). Vitamin D Metabolism and the Implications for Atherosclerosis. Ultraviolet Light in Human Health, Diseases and Environment.

[29] Sziva R., Fontányi Z., Pál É., Hadjadj L., Monori-Kiss A., Horváth E., Benkő R., Magyar A., Heinzlmann A., Benyó Z. (2020). Vitamin D Deficiency Induces Elevated Oxidative and Biomechanical Damage in Coronary Arterioles in Male Rats. Antioxidants.

[30] Fontányi Z., Sziva R., Pál É., Hadjadj L., Monori-Kiss A., Horváth E., Benkő R., Magyar A., Heinzlmann A., Benyó Z. (2021). Vitamin D Deficiency Reduces Vascular Reactivity of Coronary Arterioles in Male Rats. Curr. Issues Mol. Biol..

[31] Hadjadj L., Monori-Kiss A., Horváth E.M., Heinzlmann A., Magyar A., Sziva R.E., Miklós Z., Pál É., Gál J., Szabó I. (2018). Geometric, elastic and contractile-relaxation changes in coronary arterioles induced by Vitamin D deficiency in normal and hyperandrogenic female rats. Microvasc. Res..

[32] Pál É., Hadjadj L., Fontányi Z., Monori-Kiss A., Mezei Z., Lippai N., Magyar A., Heinzlmann A., Karvaly G., Monos E. (2018). Vitamin D deficiency causes inward hypertrophic remodeling and alters vascular reactivity of rat cerebral arterioles. PLoS ONE.

[33] Pál É., Hadjadj L., Fontányi Z., Monori-Kiss A., Lippai N., Horváth E.M., Magyar A., Monos E., Nádasy G.L., Benyó Z. (2019). Gender, hyperandrogenism and vitamin D deficiency related functional and morphological alterations of rat cerebral arteries. PLoS ONE.

[34] Sipos M., Péterffy B., Sziva R., Magyar P., Hadjadj L., Bányai B., Süli A., Soltész-Katona E., Gerszi D., Kiss J. (2021). Vitamin D Deficiency Cause Gender Specific Alterations of Renal Arterial Function in a Rodent Model. Nutrients.

[35] Sipos M., Gerszi D., Dalloul H., Bányai B., Sziva R., Kollarics R., Magyar P., Török M., Ács N., Szekeres M. (2021). Vitamin D Deficiency and Gender Alter Vasoconstrictor and Vasodilator Reactivity in Rat Carotid Artery. Int. J. Mol. Sci..

[36] Lajtai K., Tarszabó R., Bányai B., Péterffy B., Gerszi D., Ruisanchez É., Sziva R.E., Korsós-Novák Á., Benkő R., Hadjadj L. (2021). Effect of Vitamin D Status on Vascular Function of the Aorta in a Rat Model of PCOS. Oxidative Med. Cell. Longev..

[37] Murray C.D. (1926). The Physiological Principle of Minimum Work: I. The Vascular System and the Cost of Blood Volume. Proc. Natl. Acad. Sci. USA.

[38] Murray C.D. (1926). The Physiological Principle of Minimum Work Applied to the Angle of Branching of Arteries. J. Gen. Physiol..

[39] Wappler E.A., Antal P., Varbiro S., Székács B., Simon A., Nagy Z., Monos E., Nádasy G.L. (2013). Network remodeling of intramural coronary resistance arteries in the aged rat: A statistical analysis of geometry. Mech. Ageing Dev..

[40] Nádasy G.L., Szekeres M., Dézsi L., Varbiro S., Székács B., Monosa E. (2001). Preparation of Intramural Small Coronary Artery and Arteriole Segments and Resistance Artery Networks from the Rat Heart for Microarteriography and for in Situ Perfusion Video Mapping. Microvasc. Res..

[41] Török M., Merkely P., Monori-Kiss A., Horváth E.M., Sziva R.E., Péterffy B., Jósvai A., Sayour A.A., Oláh A., Radovits T. (2021). Network analysis of the left anterior descending coronary arteries in swim-trained rats by an in situ video microscopic technique. Biol. Sex Differ..

[42] Monori-Kiss A., Antal P., Szekeres M., Varbiro S., Fees A., Szekacs B., Nadasy G.L. (2020). Morphological remodeling of the intramural coronary resistance artery network geometry in chronically Angiotensin II infused hypertensive female rats. Heliyon.

[43] Hadjadj L., Várbíró S., Horváth E.M., Monori-Kiss A., Pál E., Karvaly G.B., Heinzlmann A., Magyar A., Szabo I., Sziva R.E. (2018). Insulin resistance in an animal model of polycystic ovary disease is aggravated by vitamin D deficiency: Vascular consequences. Diabetes Vasc. Dis. Res..

[44] Muldowney S., Kiely M. (2010). Vitamin D and cardiometabolic health: A review of the evidence. Nutr. Res. Rev..

[45] Kunutsor S.K., Burgess S., Munroe P.B., Khan H. (2014). Vitamin D and high blood pressure: Causal association or epiphenomenon?. Eur. J. Epidemiol..

[46] Jeong H.Y., Park K.M., Lee M.J., Yang D.H., Kim S.H., Lee S.Y. (2017). Vitamin D and Hypertension. Electrolytes Blood Press..

[47] Alemzadeh R., Kichler J., Babar G., Calhoun M. (2008). Hypovitaminosis D in obese children and adolescents: Relationship with adiposity, insulin sensitivity, ethnicity, and season. Metabolism.

[48] Veronese N., Trevisan C., Carraro S., Sarti S., Zanforlini B.M., de Rui M., Coin A., Manzato E., Sergi G. (2016). Hypovitaminosis D and fat mass in healthy older people. Eur. J. Clin. Nutr..

[49] Fry C.M., Sanders T.A.B. (2015). Vitamin D and risk of CVD: A review of the evidence. Proc. Nutr. Soc..

[50] Walsh J.S., Bowles S., Evans A.L. (2017). Vitamin D in obesity. Curr. Opin. Endocrinol. Diabetes Obes..

[51] Sacerdote A., Dave P., Lokshin V., Bahtiyar G. (2019). Type 2 Diabetes Mellitus, Insulin Resistance, and Vitamin D. Curr. Diabetes Rep..

[52] Wimalawansa S.J. (2018). Associations of vitamin D with insulin resistance, obesity, type 2 diabetes, and metabolic syndrome. J. Steroid Biochem. Mol. Biol..

[53] Warren T., McAllister R., Morgan A., Rai T., McGilligan V., Ennis M., Page C., Kelly C., Peace A., Corfe B. (2021). The Interdependency and Co-Regulation of the Vitamin D and Cholesterol Metabolism. Cells.

[54] Trechsel U., Taylor C.M., Eisman J.A., Bonjour J.P., Fleisch H. (1981). Plasma levels of vitamin D metabolites in diphosphonate-treated rats. Clin. Sci..

[55] Mirhosseini N.Z., Knaus S.J., Bohaychuk K., Singh J., Vatanparast H.A., Weber L.P. (2016). Both high and low plasma levels of 25-hydroxy vitamin D increase blood pressure in a normal rat model. Br. J. Nutr..

[56] Wilson H.D., Horst R.L., Schedl H.P. (1982). Calcium intake regulates 1,25-dihydroxy-vitamin D formation in the diabetic rat. Diabetes.

[57] Takács I., Dank M., Majnik J., Nagy G., Szabó A., Szabó B., Szekanecz Z., Sziller I., Toldy E., Tislér A. (2022). Magyarországi konszenzusajánlás a D-vitamin szerepéről a betegségek megelőzésében és kezelésében. Orv. Hetil..

[58] Halloran B.P., Barthell E.N., DeLuca H.F. (1979). Vitamin D metabolism during pregnancy and lactation in the rat. Proc. Natl. Acad. Sci. USA.

[59] Weishaar R.E., Simpson R.U. (1987). Vitamin D3 and cardiovascular function in rats. J. Clin. Investig..

[60] Xiang W., Kong J., Chen S., Cao L.-P., Qiao G., Zheng W., Liu W., Li X., Gardner D.G., Li Y.C. (2005). Cardiac hypertrophy in vitamin D receptor knockout mice: Role of the systemic and cardiac renin-angiotensin systems. Am. J. Physiol. Metab..

[61] O’Connell T.D., Berry J.E., Jarvis A.K., Somerman M.J., Simpson R.U. (1997). 1,25-Dihydroxyvitamin D3 regulation of cardiac myocyte proliferation and hypertrophy. Am. J. Physiol. Heart Circ. Physiol..

[62] Kim I.M., Norris K.C., Artaza J.N. (2016). Vitamin D and Cardiac Differentiation. Vitam. Horm..

[63] Goodwill A.G., Dick G.M., Kiel A.M., Tune J.D. (2017). Regulation of Coronary Blood Flow. Compr. Physiol..

[64] Kassab G.S., Rider C.A., Tang N.J., Fung Y.C. (1993). Morphometry of pig coronary arterial trees. Am. J. Physiol. Circ. Physiol..

[65] Tomanek R.J., Palmer P.J., Peiffer G.L., Schreiber K.L., Eastham C.L., Marcus M.L. (1986). Morphometry of canine coronary arteries, arterioles, and capillaries during hypertension and left ventricular hypertrophy. Circ. Res..

[66] Rakusan K., Wicker P. (1990). Morphometry of the small arteries and arterioles in the rat heart: Effects of chronic hypertension and exercise. Cardiovasc. Res..

[67] Zamir M., Phipps S., Langille B.L., Wonnacott T.H. (1984). Branching characteristics of coronary arteries in rats. Can. J. Physiol. Pharmacol..

[68] Zamir M. (1976). Optimality principles in arterial branching. J. Theor. Biol..

[69] Grundmann M., Haidar M., Placzko S., Niendorf R., Darashchonak N., Hubel C.A., von Versen-Höynck F. (2012). Vitamin D improves the angiogenic properties of endothelial progenitor cells. Am. J. Physiol. Physiol..

[70] Jamali N., Song Y., Sorenson C.M., Sheibani N. (2019). 1,25(OH)_2_D_3_ regulates the proangiogenic activity of pericyte through VDR-mediated modulation of VEGF production and signaling of VEGF and PDGF receptors. FASEB BioAdv..

[71] Cheung C.Y.L., Zheng Y., Hsu W., Lee M.L., Lau Q.P., Mitchell P., Wang J.J., Klein R., Wong T.Y. (2011). Retinal vascular tortuosity, blood pressure, and cardiovascular risk factors. Ophthalmology.

[72] Kahe F., Sharfaei S., Pitliya A., Jafarizade M., Seifirad S., Habibi S., Chi G. (2020). Coronary artery tortuosity: A narrative review. Coron. Artery Dis..

